# Ultrastructure of Wax-Producing Structures on the Integument of the Melaleuca Psyllid *Boreioglycaspis melaleucae* (Hemiptera: Psyllidae), with Honeydew Excretion Behavior in Males and Females

**DOI:** 10.1371/journal.pone.0121354

**Published:** 2015-03-20

**Authors:** El-Desouky Ammar, Matthew Hentz, David G. Hall, Robert G. Shatters

**Affiliations:** United States Department of Agriculture-Agricultural Research Service, Horticultural Research Laboratory, Fort Pierce, Florida, United States of America; United States Department of Agriculture, Beltsville Agricultural Research Center, UNITED STATES

## Abstract

The melaleuca psyllid, *Boreioglycaspis melaleucae* (Hemiptera: Psyllidae), was introduced to Florida as a biological control agent against *Melaleuca quinquenervia*, an invasive evergreen tree that has invaded large areas of Florida Everglades. Colonies of *B*. *melaleucae* nymphs are normally covered by white waxy secretions, and nymphs of various instars produce long bundles of white waxy filaments extending laterally and posteriorly from their abdomen. Scanning electron microscopy of ‘naturally waxed’ and ‘dewaxed’ nymphs (cleaned from wax) revealed two types of wax pore plates located dorsally and laterally on the integument of posterior abdominal segments starting with the 4th segment. Type-1 wax pore plates, with raised rim, peripheral groove, slits and pits, produce long ribbons and filaments of waxy secretions that are wound together forming long wax bundles, whereas type-2 wax pore plates, with slits only, produce shorter wax curls. Additionally, in both nymphs and adult females, the circumanal ring contained ornate rows of wax pores that produce wax filaments covering their honeydew excretions. Video recordings with stereomicroscopy showed that adult females produce whitish honeydew balls, powerfully propelled away from their body, probably to get these sticky excretions away from their eggs and newly hatched nymphs. Adult males, however, produce clear droplets of honeydew immediately behind them, simply by bending the posterior end of the abdomen downward. The possible role(s) of waxy secretions by nymphs and adults of *B*. *melaleucae* in reducing contamination of their colonies with honeydew, among other possibilities, are discussed.

## Introduction

The melaleuca tree, *Melaleuca quinquenervia* (Cav.) Blake (Myrtales: Myrtaceae), is an evergreen tree that was introduced to Florida from Australia in the late 19th century for ornamental and agro-forestry purposes. Because of its prolific reproductive capabilities, however, this tree has invaded vast areas of Florida wetlands including the Everglades [1,2]. A massive effort to restore the Everglades and to control the spread of this tree included introduction of two Australian biological control insects: the weevil *Oxyops vitiosa* Pascoe in 1997 and the melaleuca psyllid, *Boreioglycaspis melaleucae* Moore in 2002 [2,3]. The melaleuca psyllid established quickly and dispersed rapidly throughout the range of *M*. *quinquenervia* in Florida [2]. This psyllid caused high mortality of seedlings and premature leaf drop from mature trees [4,5]. Melaleuca psyllid populations spread at a rate of approximately 7 km/ year, are now widely distributed [6], and have been reported on *M*. *quinquenervia* in Puerto Rico, more than 1600 km from the nearest known release [7].

The melaleuca psyllid, *B*. *melaleucae* (Hemiptera: Psyllidae), has piercing sucking mouth parts and five nymphal instars. First instars are active but later instar nymphs are more sessile and congregate on leaves or stems, secreting copious amounts of white, waxy, threads that can completely cover older nymphs in the 3rd-5th instars [1]. Adults and nymphs were reported to feed by inserting their stylets through stomatal pores to gain access to the phloem [1]. Both stages feed on expanding buds as well as mature, fully expanded leaves. Psyllid infestation induces leaf senescence, eventually resulting in mortality of melaleuca stumps and seedlings [5,8,9]. The melaleuca psyllid can lay eggs on 27 of 42 plant species tested, but immatures developed to the adult stage only on *M*. *quinquenervia* and a few of its relatives [1].

In several psyllid species, especially tropical/subtropical ones, nymphs have evolved various mechanisms to reduce water loss from their bodies, including a protective waxy material covering at least part of their integument [10]. Morphology of the wax gland openings and waxy secretions on the integument has been studied in several aphids, mealybugs and other hemipterans, which showed variable structures among various species [11–16]. In addition to reducing water loss, waxy secretions on the integument of hemipterans are thought to limit their contact with the sticky, sugary, honeydew excreted from the same or other individuals in the colony, and possibly providing protection against fungi, parasitoids, predators, dehydration and/or frost [12,14]. We recently investigated the honeydew excretion behavior and the waxy secretions covering the honeydew in the Asian citrus psyllid, *Diaphorina citri*, vector of the economically important citrus greening (huanglongbing) bacterium [17,18]. In the present work, we report ultrastructural studies on the wax-producing structures, as well as honeydew excretion behavior, in the melaleuca psyllid *B*. *melaleucae*, and compare them with those of *D*. *citri* and other hemipterans.

## Materials and Methods

### Melaleuca Psyllids Used

Most of the melaleuca psyllid (*B*. *melaleucae*) individuals studied here were collected in March-May, 2012 and 2013, from melaleuca trees (*M*. *quinquenervia*) growing in an area privately owned by M. Hentz in Port St. Lucie, South Eastern Florida. Nymphs and adults of *B*. *melaleucae* were brought to the laboratory with small terminal shoots of melaleuca in plastic bags, and were then kept for observation and stereomicroscopy in plastic Petri dishes for a few to several days before preparing some of them for SEM. The Petri dishes were placed on the bench top in the laboratory at 23.7 ±1.5°C with 14 h light per day. Additionally, in September 2014, Dr. Paul D. Pratt (USDA-ARS, Fort Lauderdale, FL) kindly supplied us with a colony of melaleuca psyllid nymphs and adults reared in his laboratory. This colony was caged on a small melaleuca plant (ca. 50 cm high), kept in the laboratory under the above conditions for several weeks, and used for some of the behavioral studies mentioned below.

### Stereomicroscopy and Scanning Electron Microscopy (SEM)

Eggs, nymphs and adults of the melaleuca psyllid were initially examined and photographed using a stereomicroscope (Leica MZ16) fitted with a Leica DFC 320 camera (Leica, Switzerland). Later, adults and nymphs were examined by SEM using the following three preparation methods. In the first method, used to study wax structures on the cuticle surface, insects were immobilized by freezing at −20°C for a few minutes, then directly mounted on SEM stubs as mentioned below without fixation or dehydration; specimens examined by this method will be referred to as ‘waxed’ since their waxy secretions were still intact and in their natural position “*in situ*”. The second method, used for studying the wax-producing structures on the cuticle surface or on the circumanal ring (around the anus), insects were ‘dewaxed’, i.e. cleaned from their natural waxy secretions by a method modified from Lucchi and Mazzoni [15]. This was done by soaking insects either in 100% chloroform or acetone in a small glass cavity-dish (cavity ca. 20-mm diam., 5 mm deep) covered with a glass slide. Insects were soaked in either of these solvents overnight at room temperature in a fume hood, followed by immersion of the samples in 70% then 100% ethanol for 1 h each, and finally in hexamethyldisilazane (HMDS, 2 changes, 1–2 h each) before air drying. The third method was similar to the second except that the HMDS step was eliminated.

Following any of the three preparation methods mentioned above, insects were mounted, under a stereomicroscope, on black conductive double-sided adhesive discs (9–12 mm diameter) placed on aluminum stubs (SPI Supplies, West Chester, PA), using fine-pointed forceps (Fontax no. 5; Electron Microscopy Sciences, Washington, PA). Mounted specimens were then sputter coated with Gold-Palladium for 120 sec using Hummer 6.2 Sputter Coater (Anatech USA, Union City, CA). Coated specimens were then examined at 5 or 10 Kv using a scanning-transmission electron microscope (Hitachi S-4800, Hitachi, Pleasanton, CA) in the SEM mode at magnifications of 100x to 10,000x. The number of melaleuca psyllid individuals examined by SEM, from each of the ‘waxed’ and ‘dewaxed’ categories, were 9–13 younger nymphs (1st-3^rd^ instar), 18–19 older nymphs (4^th^-5^th^ instar), and 17–18 adults (males and females). Approximate identification of psyllid nymphal instars was based on body size and relative size of wing pads as described previously for *D*. *citri* [19, 20]. Morphological terms used for describing the anal area in nymphs, males and females are based on those used by Brittain [21] for the pear psylla, *Psylla mali*.

### Honeydew Excretion Behavior in Adult Males and Females

Honeydew excretion behavior of melaleuca psyllid males and females was observed and photographed using a stereomicroscope (Leica M60) fitted with a video camera (Leica DFC290 HD) (Leica, Switzerland). Groups of 4–5 males and/or females were caged on excised leaves or a piece of terminal young shoot of melaleuca in a 9 cm clear plastic Petri dish. Several video recordings (20–60 min each) of honeydew excretion behavior of males or females were undertaken either directly or through the cover of the Petri dish, while these males or females were feeding on the leaves and producing their honeydew excretions. S1 Video provided here (ca. 2 min), is composed of eight short clips showing four females (clips 1–6) followed by two males (clips 7 and 8), each producing honeydew excretion balls or droplets. These clips were recorded at normal (real time) speed but were cut, assembled and are played back here at a much lower speed (16x slower for females and 4x slower for males), to show the excretion behavior in both sexes more clearly. Windows Movie Maker program (v. 2.6) was used to cut the original clips and assemble this video.

## Results

### Melaleuca Psyllid Colonies and Waxy Secretions

**Fig 1:**
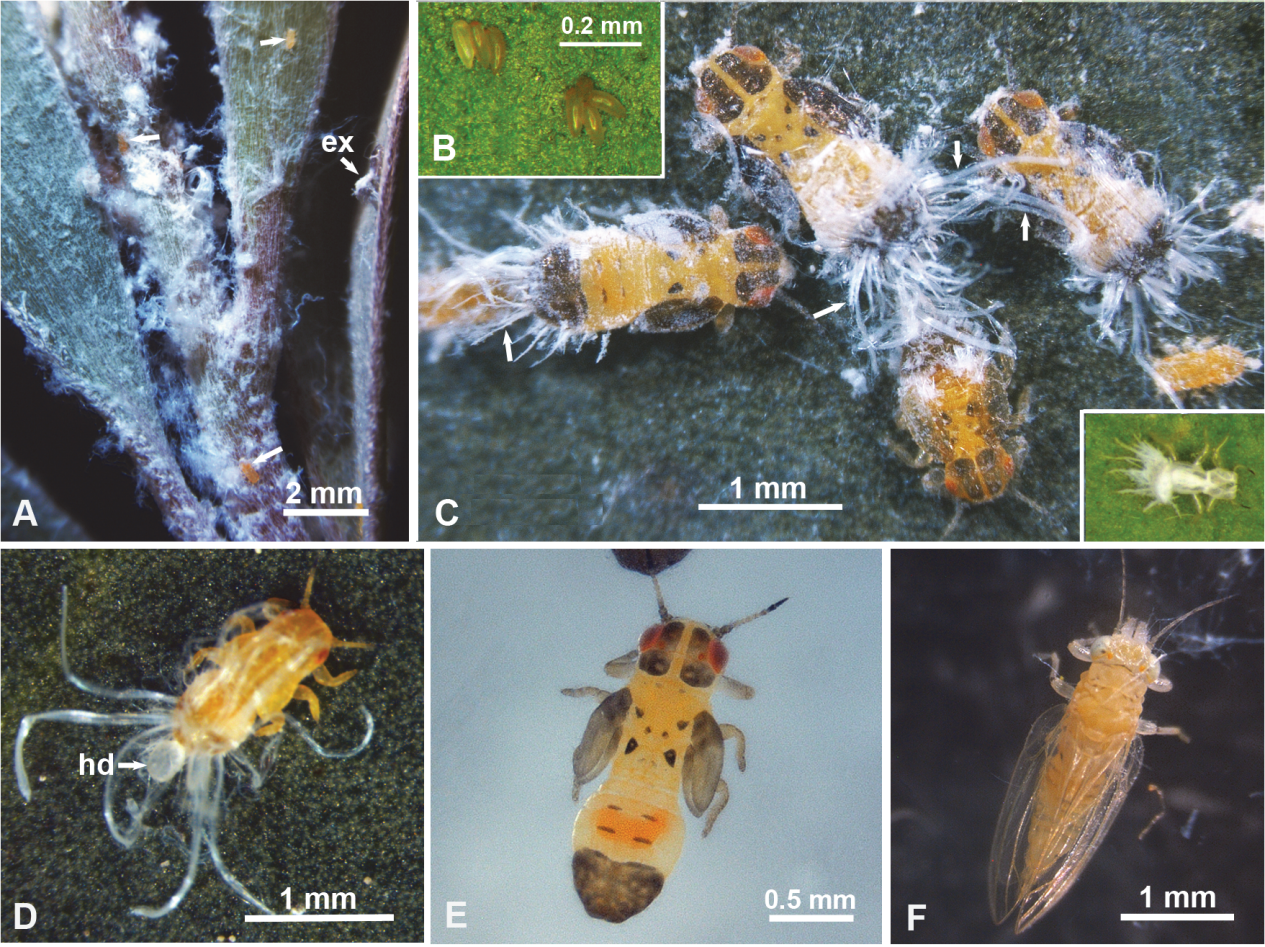
Melaleuca psyllid colonies, eggs, nymphs, adults and waxy secretions. **A**. White waxy secretions covering a colony of nymphs on a terminal shoot of melaleuca; ex, exuvia; unlabeled arrows indicate nymphs. **B.** Egg clusters, with their stalks embedded in leaf tissues. **C**. Nymphs of various instars with long wax bundles (arrows) extending laterally and posteriorly from their abdomens; inset shows an exuvia attached to the leaf with wax bundles still attached to it. **D.** Younger (2^nd^ instar) nymph producing a ball of honeydew (hd) covered with a thin layer of whitish waxy material. **E.** Older (4^th^-5^th^ instar) nymph after ‘dewaxing’ (cleaning from wax by immersion in chloroform). **F.** Adult male (not dewaxed).

Colonies of the melaleuca psyllid, *B*. *melaleuca*, were found mainly on young terminal shoots of melaleuca trees. These colonies were distinguished by white, fluffy, waxy secretions, covering various nymphal instars, especially on young tender stems or at the axillary corners between young terminal shoots and young leaves (Fig. 1A). Melaleuca psyllid eggs, laid singly or in small clusters by the females, are yellowish, spindle shaped, and their stalks embedded in the leaf tissues (Fig. 1B). Nymphs of various instars usually had long bundles of white waxy material extending laterally and posteriorly from their abdomen, and these bundles increased in length, thickness and density in older instars (Fig. 1C, 1D). These wax bundles were still attached to the exuvia after molting (Fig. 1C, inset). No similar wax bundles were observed to originate from the head or thorax of nymphs (Fig. 1C, 1D) or from any part of the body of adult females or males (Fig. 1F). Wax bundles could be removed by ‘dewaxing’ the nymphs in chloroform or acetone as mentioned earlier. The dewaxing procedure used did not seem to change the color of nymphs or adults appreciably. Before and after dewaxing, younger (1^st^-2^nd^ instar) nymphs are orange colored with red eyes (Fig. 1D), whereas older (4^th^-5^th^ instar) nymphs have yellow-orange body with brown wing pads, brown abdominal tip and several brown marks on the head, antenna, thorax and abdomen (Fig. 1C, 1E). Adults, however, are yellow-orange in color, with transparent wings and some brown stripes dorsally on the abdomen (Fig. 1F).

### Ultrastructure and Distribution of Wax and Wax Pore Plates in Nymphs

**Fig 2:**
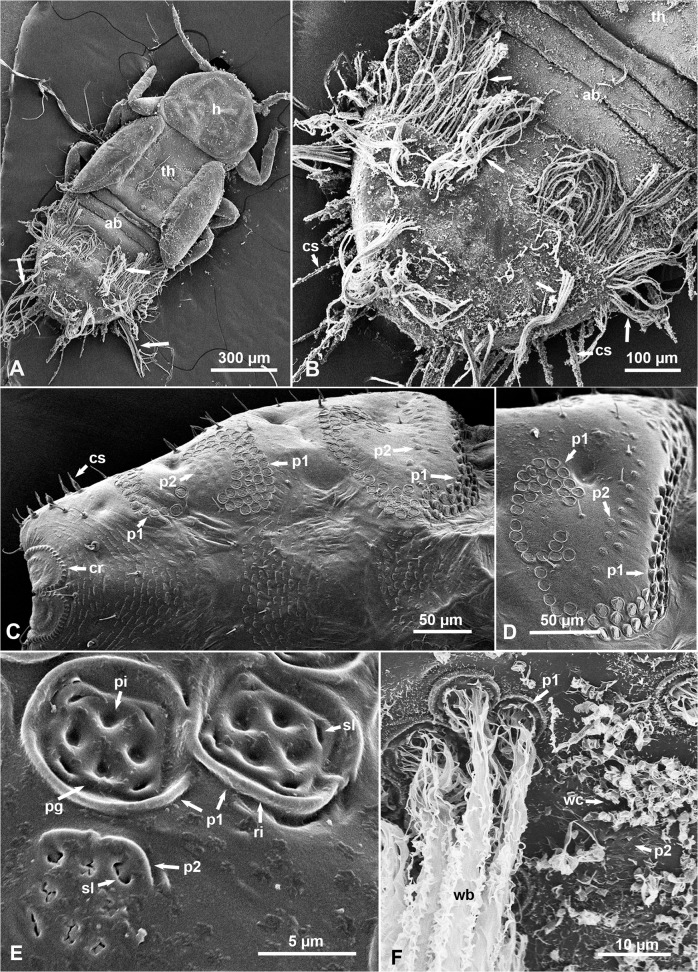
SEM of waxy secretions and wax pore plates on the integument of melaleuca psyllid nymphs (dorsal views). **A & B.** Older (4^th^-5^th^ instar) nymph with wax bundles (arrows) extending from the abdomen (ab); note that the head (h), thorax (th) and first 3 abdominal segments do not have wax bundles. **C&D**. Part of the abdomen in ‘dewaxed’ 4^th^-5^th^ instar nymphs, showing arrays of wax pore plates of types 1 (p1) and 2 (p2), as well as the circumanal ring (cr) at the posterior end of the abdomen. **E.** Higher magnification of wax pore plates, types 1 (p1) and 2 (p2) in dewaxed nymphs; note the open slits (sl) in both types, and the pits (pi), peripheral groove (pg) and raised rim (ri) especially in type-1. **F.** Wax bundles (wb) coming out of type-1 pore plates (p1), and wax curls (wc) apparently coming out of type-2 pore plates (p2), on the abdomen of a waxed nymph. Additional abbreviations: cs, circumabdominal setae.

SEM examination of melaleuca psyllid nymphs of various instars revealed no wax bundles or wax producing structures on the thorax or the first three abdominal segments (Fig. 2A, 2B). However, on the rest of the abdominal segments, dorsally and laterally starting with the 4^th^ segment several arrays of wax pore plates were found (Fig. 2C, 2D). These wax pore plates can be divided, according to their ultrastructure, into two types designated here as types 1 and 2. Both types of pore plates are quadrate or quasi-spherical in shape, with a mean diameter of 7.37 ± 0.84 μm (N = 84), and each contained 4–8 apparently open slits (straight, curved or star-shaped) that averaged 1.68 ± 0.30 μm in length (Fig. 2E). However, type-1 pore plates are distinguished by a raised rim and a deep peripheral groove on the inside of this rim, whereas type-2 pore plates themselves were slightly raised above the cuticle, without a complete rim or only with a partial one (Fig. 2C-2E). Type-1 pore plates also usually contained four deep pits in addition to the open slits found in both types 1 and 2 (Fig. 2E). By comparing waxed and dewaxed nymphs, it can be concluded that type-1 pore plates produced long ribbons and filaments of waxy secretions that appeared to be coming out of the peripheral groove, open slits and/or pits, and to be eventually wound together forming very long wax bundles that extend laterally and posteriorly from the abdomen (Fig. 2A, 2B, 2F). Type-2 pore plates, however, appeared to produce shorter curls of wax from their slits (Fig. 2F).

In older (4^th^-5^th^ instar) nymphs, at least three straight or curved (C-shaped) arrays that included both types-1 and -2 pore plates were found dorso-laterally on each side of the abdomen (Fig. 2C, 2D). Each array is composed of 3–5 rows of type-1 pore plates under which (posteriorly) are 1–3 rows of type-2 pore plates (Fig. 2C, 2D). However, in younger (1^st^-2^nd^ instar nymphs) only two arrays with fewer rows of pore plates in each were normally found (data not shown). The majority of type-1 and type-2 pore plates faced either dorsally or laterally, but type-1 pore plates of the anterior-most array normally faced anteriorly, towards the head, and thus, their rims are raised much higher than those of other type-1 pore plates (Fig. 2C, 2D).

### Circumanal Ring and Circumabdominal Setae in Nymphs

**Fig 3:**
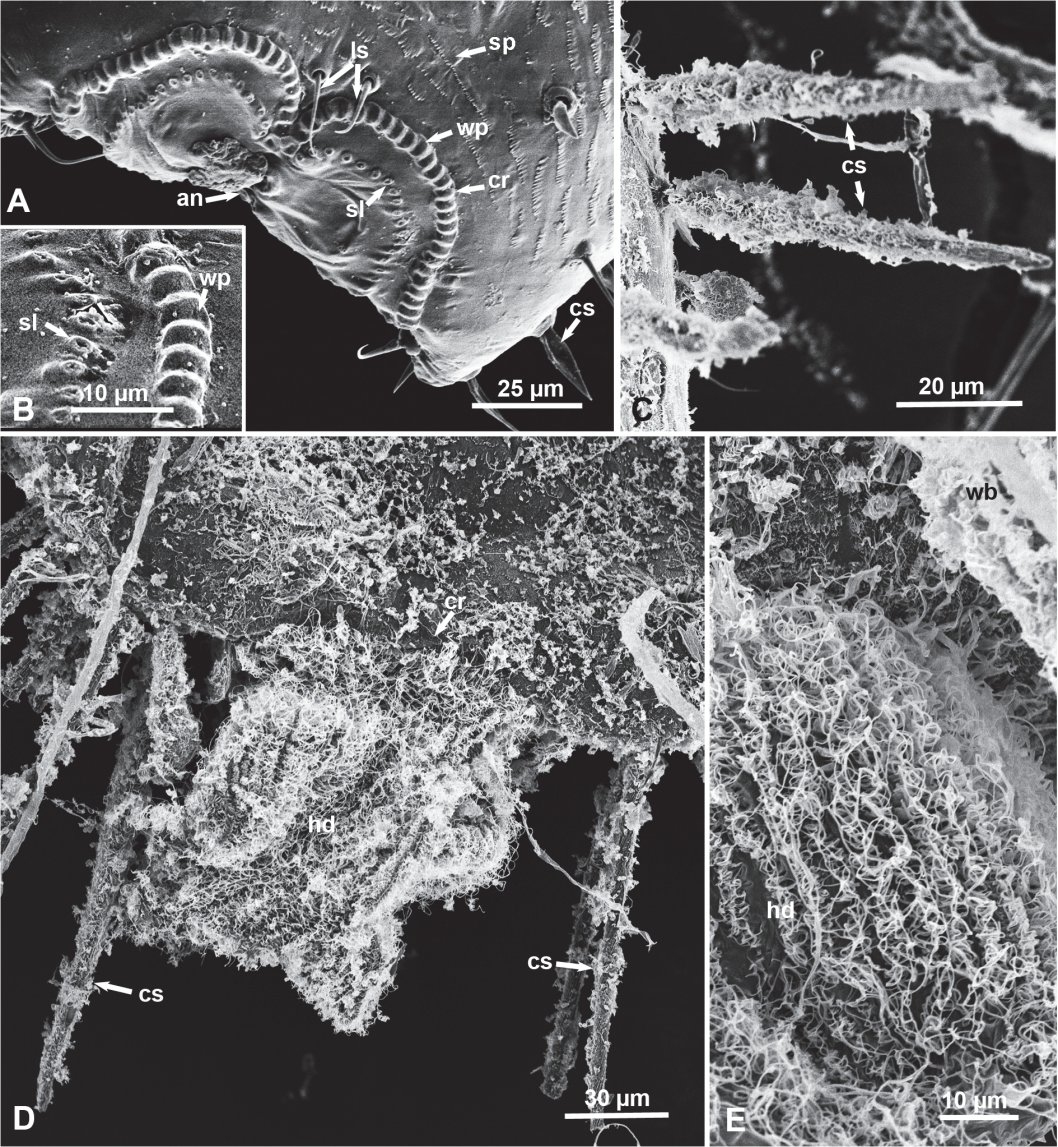
SEM of the circumanal ring, honeydew excretions and circumabdominal setae of melaleuca psyllid nymphs (dorsal views except in D). **A & B.** Part of the circumanal ring (cr) around the anus (an) in dewaxed nymphs, with an outer row of wax pores (wp) and an inner row of slits (sl); ls, long setae; sp, rows of short spines pointed posteriorly. **C.** Circumabdominal setae (cs) covered with wax filaments in a ‘waxed’ nymph. **D.** Ventral view of the posterior end of the abdomen in a 2^nd^-3^rd^ instar nymph with wax-covered honeydew (hd); cr, circumanal ring. **E.** Higher magnification of wax filaments covering honeydew excretions (hd) at the end of the abdomen; wb, wax bundles. Additional abbreviations: cs, circumabdominal setae.

In the melaleuca psyllid nymphs of all instars the anus is found at the posterior tip of the abdomen, surrounded by an ornate circumanal ring (Fig. 3A) parts of which can usually be seen dorsally as well as ventrally. Ultrastructurally, using SEM, the circumanal ring is composed of an undulating row of wax pores, each 4.34 ± 0.40 μm long and 1.74 ± 0.20 μm wide, with cuticular ridges 0.50 ± 0.11μm wide, between them (Fig. 3A, 3B). Inside this row of pores, another row of much smaller deep slits, each 1.31± 0.17 μm wide, is found (Fig. 3A, 3B). In some ‘waxed’ nymphs (uncleaned from wax), whitish honeydew balls were still attached to their posterior end (Figs. 1C, 3D). On the surface of these honeydew excretions, several lines of fine convoluted wax filaments appeared to be coming out of the wax pores and/or slits of the circumanal ring (Fig. 3D, 3E).

Two long setae, pointed posteriorly, were found dorsally just anterior to the center of the outer row of wax pores of the circumanal ring (Fig. 3A). Slightly shorter setae, also pointed posteriorly, were scattered dorsally on other abdominal segments (Fig. 2C). Additionally, multiple rows of short spines, pointed posteriorly, were found dorsally in the abdominal segments with wax pore plates (Fig. 3A). Furthermore, strong circumabdominal setae, mostly pointed posteriorly, were found laterally around the abdomen (Figs. 2A-C, 3A, 3C-D). These setae were normally covered with wax filaments, (Fig. 3C, 3D) that could be cleaned with chloroform or acetone (Figs. 2C, 3A). The circumabdominal setae increased in size and number in older compared to younger nymphs, but they were not found in adult males or females.

### Circumanal Ring, Ovipositor and Wax-Producing Structures in Females

**Fig 4:**
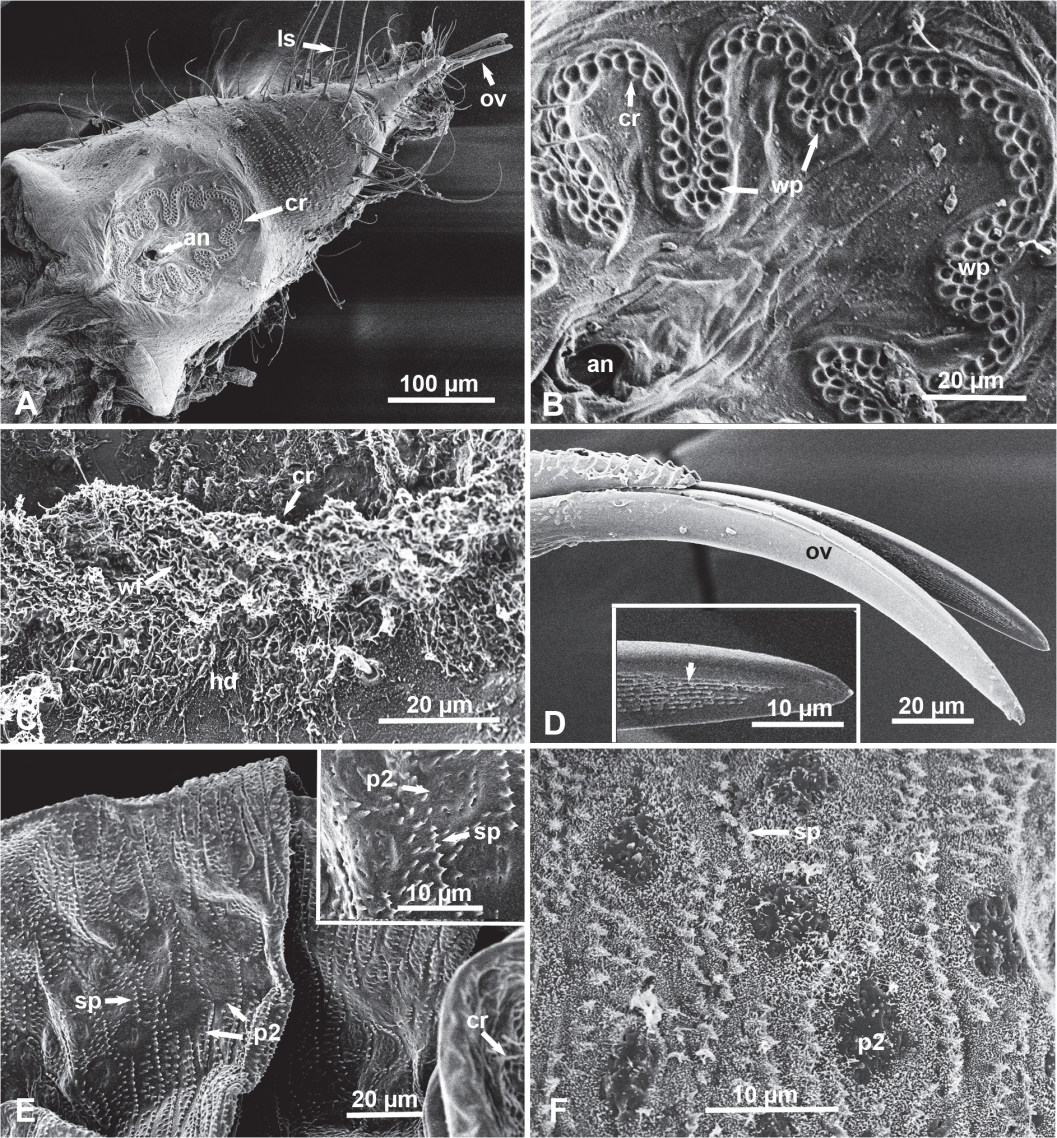
SEM of adult females of the melaleuca psyllid at or near the posterior end of the abdomen. **A & B.** Dorsal views of the circumanal ring (cr) around the anus (an) in a ‘dewaxed’ female, with the ovipositor (ov) and several long setae (ls) near the tip of the abdomen; note the divided-leaf like morphology of the circumanal ring (cr) and its double or multiple rows of wax pores (wp). **C**. Part of the abdomen’s end in a ‘waxed’ female, showing wax filaments (wf) oozing out of the circumanal ring (cr) and covering honeydew excretion (hd). **D.** Dorso-lateral view of the two ovipositor valvulae (ov), and details of their inner side (inset) showing several rows of fine ridges (arrow). **E.** Lateral part of the two abdominal terga anterior to the circumanal ring (cr) in a ‘dewaxed’ female showing type-2 wax pore plates (p2), with higher magnification in the inset; note multiple rows of short spines (sp) pointed posteriorly. **F**. Similar area to that in E, but in a ‘waxed’ female, showing type-2 wax pore plates (p2), with fine tufts of wax covering the terga and spines (sp).

As is the case with nymphs, adult females, but not males, of the melaleuca psyllid possessed a circumanal ring around the anus. In the females, however, the shape of the circumanal ring was different from that in nymphs (Figs. 3A, 4A, 4B). In the female, this ring was shaped like a divided multi-lobed leaf, which was seen only on the dorsal side near the posterior end of the abdomen (Fig. 4A, 4B). Ultrastructurally, using SEM, the circumanal ring of the female was composed of 2–4 compact rows of wax pores, each pore is quasi-spherical or oblong, 2.72 ± 0.37μm long, and 2.22 ± 0.50 μm wide, with raised ridges (0.39 ± 0.11 μm wide) between them (Fig. 4B). In waxed females (uncleaned from wax), convoluted wax filaments were observed apparently coming out of these pores (Fig. 4C). At the end of the female’s abdomen, the two pointed valvulae of the ovipositor were slightly serrated near the end, and they had several longitudinal rows of very fine ridges on their inner sides (Fig. 4D and inset). Several long hairs/setae, mostly pointed posteriorly, were found near the tip of the abdomen, especially between the circumanal ring and the ovipositor (Fig. 4A).

In addition to the wax pores described on the circumanal ring, adult females also possessed a few arrays of type-2 wax pore plates (diameter = 6.58 ± 1.02 μm) on both sides of the two abdominal terga anterior to the circumanal ring. (Fig. 4E and inset). Around these pore plates, these terga were almost covered with multiple rows of short cuticular spines, all pointed posteriorly (Fig. 4E and inset). In waxed females, very short tufts of wax filaments covered these terga and their spines; some fine filaments were found also on the slits of these type 2 pore plates (Fig. 4F).

### Anal Tube, Aedeagus and Wax-Producing Structures in Males

**Fig 5:**
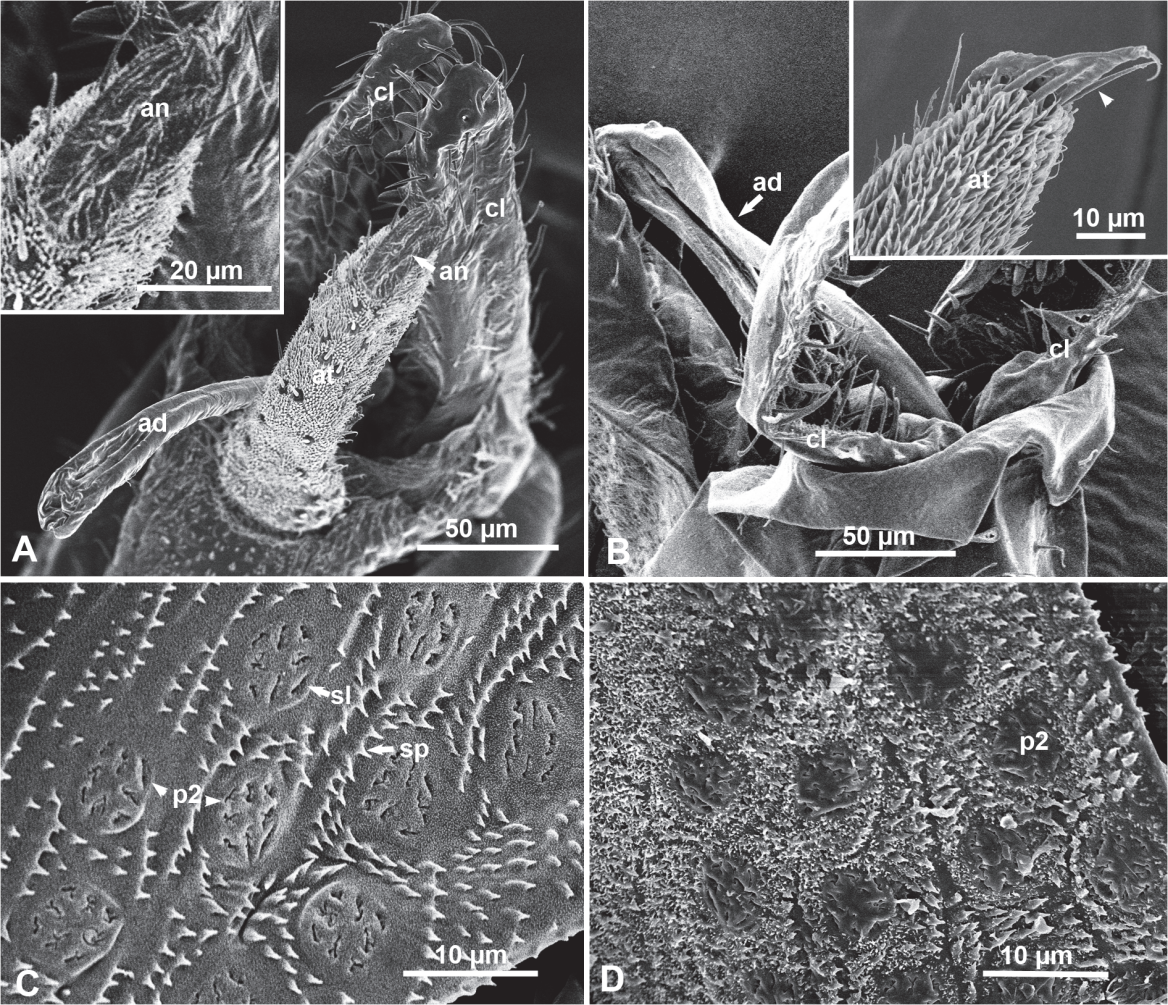
SEM of adult males of the melaleuca psyllid at or near the posterior end of the abdomen. **A.** Dorsal view of the anal tube (at), anus (an), aedeagus (ad) and two claspers (cl); the inset shows details of the anus (an), lacking a circumanal ring like that of the female (compare with Fig. 4A, 4B). **B**. Ventro-lateral view of the folded aedeagus (ad) and two claspers (cl); inset shows lateral view of tip of the anal tube (at) covered with thick hair, and several long spines (arrowhead) around the anus. **C.** Lateral part of an abdominal tergum anterior to the anal plate in a ‘dewaxed’ male showing type-2 pore plates (p2); sl, slits; sp, rows of short spines pointed posteriorly. **D.** Similar area to that in C, but in a ‘waxed’ male, showing type-2 pore plates (p2) and fine wax tufts covering the tergum and its spines.

The anal opening in males is found dorsally at the end of a long anal tube covered with fine thick hairs, near the posterior end of the abdomen (Fig. 5A, 5B). The anus is a long oval slit, surrounded by long hairs, but is lacking a circumanal ring or wax pores like those described in nymphs or adult females (Fig. 5A, 5B, and their insets). The long jointed aedeagus, normally folded when not in use for mating, is housed between the anal tube and two spiny claspers at the tip of the abdomen (Fig. 5A, 5B). In males, as is the case with females, a few arrays of type-2 wax pore plates (diameter = 6.83± 0.45 μm) were found on both sides of the two abdominal terga anterior to the anal plate (Fig. 5C). Also as the case with females, these terga were almost covered with multiple rows of short cuticular spines, all pointed posteriorly (Fig. 5C). In waxed males, very short tufts of wax filaments covered these terga, their spines. Some tufts were found also on the slits of these type 2 pore plates, suggesting that these tufts may come out of these pore plates (Fig. 5D).

### Head, Mouth Parts and Antennae of Nymphs and Adults

**Fig 6:**
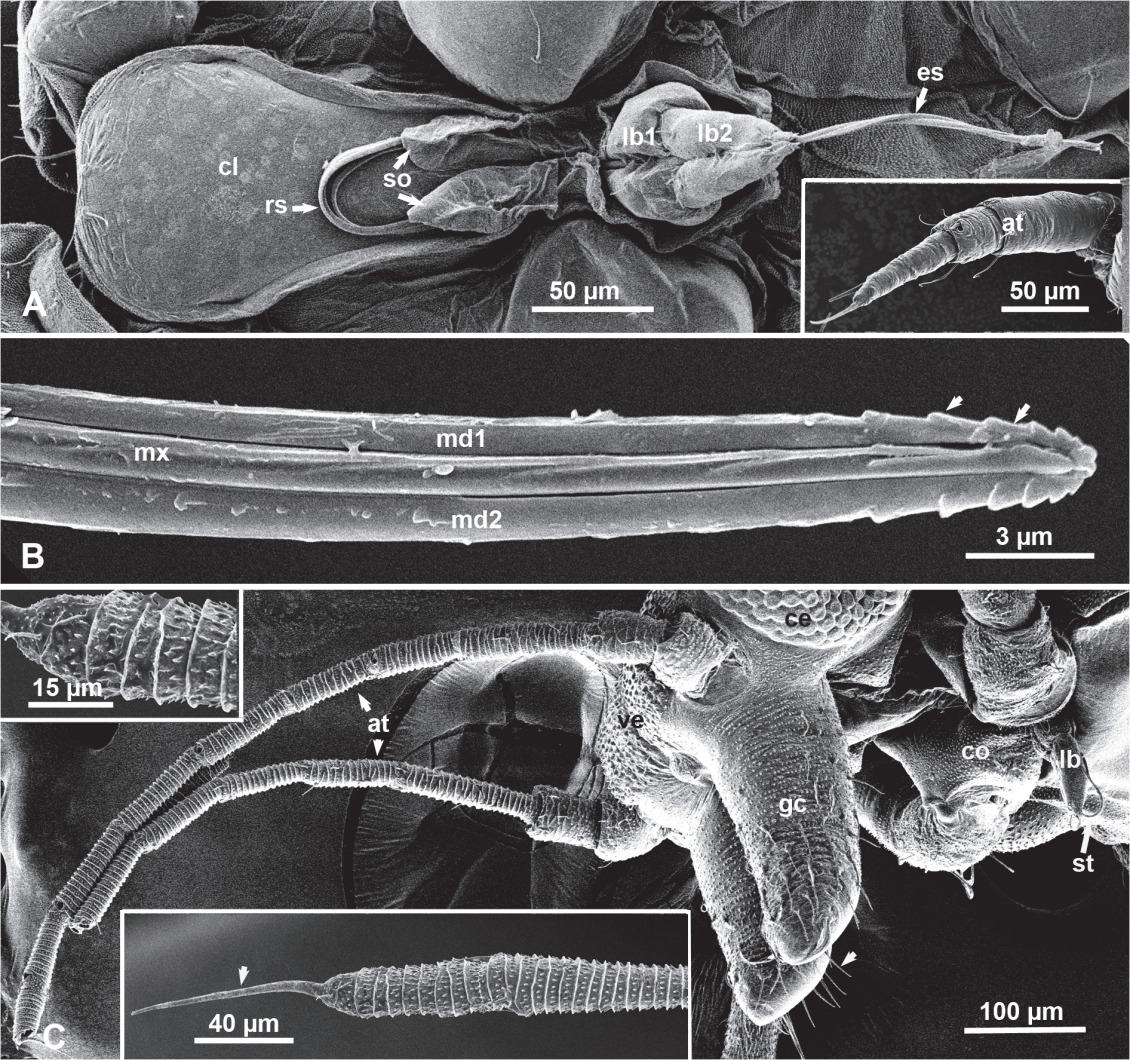
SEM of the head and mouth parts of nymphs and adults of the melaleuca psyllid. **A**. Ventral view of part of the head and mouth parts in a ‘dewaxed’ nymph, showing the clypeus (cl), two segments of the labium (lb1, lb2), extended part of the stylet bundle (es); retracted part of the stylets (rs) is looped behind the paired stylet-holding organ (so); inset shows nymphal antenna (at). **B.** Lateral view of terminal part of the stylet bundle, showing the (inner) maxillary stylets (mx), and serration (arrows) near the tip of the (outer) mandibular stylets (md1, md2). **C.** Ventro-lateral view of the head and mouth parts of an adult, showing the antenna (at), vertex (ve), compound eye (ce), two genal cones (gc) with long hairs/setae (arrowhead), partly extended stylets (st), and the labium (lb), between the two front coxae (co); upper and lower insets show details of distal segments of the antenna encircled with rows of short spines, with a long pointed bristle (arrowhead) at the end of the terminal segment.

No wax or wax producing structures were detected on the head, mouth parts or antennae of nymphs, adult males or females of the melaleuca psyllid. The mouth parts of nymphs are composed of a two-segmented labium, with a long median groove in which part of the partially or fully extended stylet bundle is normally housed (Fig. 6A). The distal part of the outer (mandibular) stylets of the stylet bundle is finely serrated in both nymphs and adults (Fig. 6B). No labrum was detected in the nymphs, but a paired organ for holding the retracted part of the stylet bundle against the lower/posterior part of the clypeus was observed (Fig. 6A). This organ is absent from the adults, where the retracted stylets cannot be seen externally (Fig. 6C).

The antennal flagellum in adults has 8 segments, each with several circular rows of very short spines, but the distal segment ends with a very long pointed bristle (Fig. 6C and its insets). In nymphs, the antennal flagellum had only three segments with no short spines, but with a few long hairs/setae at the distal end of each segment, and two long bristles at the end of the distal segment (Fig. 6A inset).

### Honeydew Excretion Behavior in Adult Males and Females

**Fig 7:**
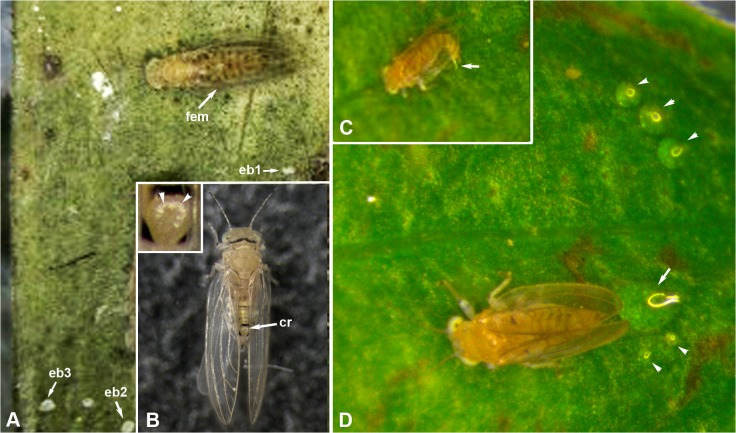
Honeydew excretion behavior in melaleuca psyllid adults of both sexes. **A**. Still image (taken from S1 Video), showing one female (fem, #4 in that video) with three excretion balls (eb1, eb2 and eb3) that were produced consecutively in the three clips shown in S1 Video (note the angle and distance between the female body and these 3 excretion balls). **B.** Melaleuca psyllid female at the beginning of the honeydew excretion process, with a tiny circle of waxy secretions (arrowheads in the inset) coming out of the openings of its circumanal ring (cr) near the posterior end of the abdomen. **C&D**. Still images taken from video, showing a male (in C) bending the posterior end of its abdomen downward to place a drop of honeydew (arrow) on the leaf surface, and the same male (in D) with several clear drops of honeydew excretions around it (arrowheads), with a larger drop (arrow) immediately behind its abdomen (note reflected light on each drop).

The melaleuca psyllid males and females produced their honeydew excretion balls/droplets as shown in Fig. 7 and S1 Video. For females, the time that elapsed between producing these balls was about 16–25 min or longer, during which a whitish-colored excretion ball could be seen growing gradually in size at the anal opening dorsally near the posterior end of the abdomen. Whitish waxy secretions were observed to exude from the circumanal ring during formation of the excretion ball (Fig. 7B and inset). When about to dislodge this ball, the female starts by twitching its wings briefly, raising the end of its abdomen upward, then pushing its excretion ball dorsally through the pyramid-shaped folded wings. In some cases, she leaves this ball momentarily held between the wings, while she bends the abdomen downward, then uses the end of the abdomen (with a strong thrust upward at a very high speed) to propel the ball upward and sideways. This excretion ball normally did not fall immediately behind the female body, but usually it fell at an angle to the left or right sides of the female body about 1–3 body lengths away (Fig. 7A and S1 Video). Males, however, simply bent the posterior end of their abdomen downward and laid a colorless drop of honeydew immediately behind or under the end of the abdomen (Fig. 7C, 7D and S1 Video). Apparently, when each drop became too big they moved to another nearby feeding site and produced another one (Fig. 7D).

## Discussion

The melaleuca psyllid, *B*. *melaleucae*, proved to be quite different with regard to the ultrastructure and distribution of wax and wax-producing structures, from two previously studies psyllid species: the mulberry psyllid, *Anomoneura mori* [22], and the Asian citrus psyllid, *D*. *citri* [17]. In the melaleuca psyllid nymphs an extensive system of wax pore plates has been found that produces long wax bundles extending dorsally and laterally from the abdomen, which is not the case with either *A*. *mori* or *D*. *citri*. In the latter two species, visible waxy secretions seem to be produced only by the circumanal ring in nymphs and female adults only to cover their honeydew excretions [17,22].

Several hemipteran species, especially among aphids (Aphididae), whiteflies (Aleyrodidae), mealybugs (Pseudococcidae), scale insects (Coccidae) and planthoppers (Fulgoroidea) produce waxy secretions to cover all or part of their integument [12–15, 23,24]. Wax glands in Hemiptera are composed of greatly enlarged epidermal cells underlying a modified cuticle that forms distinctive wax pores or pore plates which are different in shape and structure between various species [14]. Secreted wax in the form of threads or fine sheets pass out of the cuticle as filaments, the arrangement of which in the cuticle above each epidermal cell gives rise to distinctive wax forms for each species [11,12].

Both types-1 and-2 wax *pore plates*, described here for *B*. *melaleucae*, are quite different from types-1 and-2 wax pores reported earlier on the integument of the woolly oak aphid, *Stegophylla brevirostris* Quednau [16]. Both of these types of wax pores in *S*. *brevirostris* had peripheral grooves similar to those found in this study, but no slits or pits like those described here for types-1 and 2 pore plates of *B*. *melaleucae*. Also, the latter were very different from the wax pore plates described on the integument of the beech woolly aphid, *Phyllaphis fagi* [12,14], the flatid planthopper, *Metcalfa pruinosa* [15], the multilocular wax pores described for some scale insects [25], or the trilocular pores described for the mealybug *Maconellicoccus hirsutus* [13]. With the beech woolly aphid, *P*. *fagi*, Smith [14] indicated that from the annular depression of each *pore* plate exuded fine wax threads that together make up the hollow ‘skien’ produced by each wax gland cell. These ‘annular depressions’ seem to be similar to the ‘peripheral grooves’ of type-1 *pore* plates that we described here for *B*. *melaleucae*. The latter species, as we have shown, produces long filaments of wax that are wound together to form large bundles extending laterally and posteriorly, dorsally or dorso-laterally, on the posterior part of the abdomen.

Feeding on the phloem presents some challenges to hemipteran insects. The very high sugar content and osmotic pressure of phloem sap is countered by sucrase-transglucosidase activity in their guts, which transforms excess sugar into long-chain oligosaccharides voided as honeydew excretion [26]. This type of excretory process presents another problem for these insects: how to avoid being contaminated or even drowned by their own sticky excreta, especially for the more vulnerable eggs and young nymphs [27]. The nymphs and female adults of almost all psyllid species morphologically studied in details by light or electron microscopy, so far, were shown to have a circumanal ring (around the anus) that contains wax pores producing wax filaments around their honeydew excretions, although such wax pores may be ultrastructurally different between species. These psyllid species include the mulberry psyllid *A*. *mori* [22], the apple psyllid *Psylla mali* [21], the Asian citrus psyllid *D*. *citri* [17] and the melaleuca psyllid *B*. *melaleucae* (this study). These psyllid species also were reported to have somewhat similar wax-covered (circumabdominal) setae around the abdomen in nymphs but not in adults. Interestingly, an African psyllid species, *Diaphorina enderleini* (the nymphs of which do not have a functional circumanal ring and do not produce wax) was found to be involved in an unusual mutualistic interaction with two ant species apparently to help with getting rid of its sticky (non-wax covered) honeydew excretions [28].

Although the ultrastructural morphology of the wax pores in the circumanal ring of nymphs and females are different between *D*. *citri* and *B*. *melaleucae*, those in the nymphs of both species are somewhat similar in having an outer row of wax pores and an inner row of deep/open slits [17]. It is clear that in both species wax pores and/or slits of the circumanal ring produce wax filaments that cover their honeydew making it less sticky, and thus, along with the circumabdominal setae, help in minimizing contamination of nymphs with the thick sugary honeydew. In both *B*. *melaleucae* and *D*. *citri*, only the females were found to propel their wax-covered honeydew pellets/balls away from their bodies, a behavior likely to protect their eggs and newly hatched nymphs from honeydew contamination ([17] and present study). It is worth noting that adult males of both *D*. *citri* and *B*. *melaleucae* do not have a cicrcumanal ring, do not produce wax to cover their honeydew excretions, and do not propel their honeydew excretions away from their bodies ([17] and present work). It would be interesting to see if this difference in honeydew excretion behavior between males and females is universal among other psyllid species.

Infrared microscopy and mass spectroscopy revealed that, in addition to various sugars, honeydew excretions of *D*. *citri* nymphs and females are covered with a thin layer of wax similar in profile to ester waxes [17]. Chemical analysis also indicated that wax esters are major constituents of the external waxes produced by the woolly oak aphid, *S*. *brevirostris*, [16] and the giant whitefly, *Aleurodicus dugesii* [29]. Preliminary chemical analysis of waxy secretions produced by the melaleuca psyllid nymphs suggested that they contain a mixture of paraffin hydrocarbons and wax esters that are currently under further chemical investigation (R. Alessandro and E.-D. Ammar, unpublished). Pope [12] argued that since considerable energy must be expended to produce external waxes by hemipteran insects, they must derive some ‘help or protection’ from the varied wax coating they produce. This ‘help’ may include water proofing, being less obvious to natural enemies, avoiding honeydew contamination, and may also have an ‘anti-molestation’ function against parasites or predators [12]. Nelson et al. [29] also indicated that, with the giant whitefly, the external wax filaments appear to form a barrier that would be difficult for parasitoids and predators to penetrate to attack either immature stages or adults. Thus, in addition to the possible role of the integument wax of the melaleuca psyllid in limiting the contamination of nymphs with honeydew, other possible roles of this integument wax including protection from water loss, adverse weather conditions and/or natural enemies, require further investigation.

## Supporting Information

S1 Video Honeydew excretion behavior of melaleuca psyllid adults. This video (approximately 2 min) is assembled from eight short clips showing females #1–3 (clips 1–3), then female #4 (clips 4–6), followed by two males (clips 7 & 8). All clips were recorded at real time (normal speed) and are played back at a much slower speed (1/16th their normal speed for females and 1/4th their normal speed for males).
(WMV)

## References

[1] Purcell MF, Balciunas JK, Jones P (1997) Biology and host-range of *Boreioglycaspis melaleucae* (Hemiptera: Psyllidae), potential biological control agent for *Melaleuca quinquenervia* (Myrtaceae). Environmental Entomology 26: 366–372.

[2] Chiarelli RN, Pratt PD, Silvers CS, Blackwood JS, Center TD (2011) Influence of temperature, humidity, and plant terpenoid profile on life history characteristics of *Boreioglycaspis melaleucae* (Hemiptera: Psyllidae), a biological control agent of the invasive tree *Melaleuca quinquenervia* . Annals of the Entomological Society of America 104: 488–497.

[3] Center TD, Purcell MF, Pratt PD, Rayamajhi MB, Tipping PW, et al (2012) Biological control of *Melaleuca quinquenervia*: an Everglades invader. Biocontrol 57: 151–165.

[4] Franks SJ, Pratt PD, Tsutsui ND (2011) The genetic consequences of a demographic bottleneck in an introduced biological control insect. Conservation Genetics 12: 201–211.

[5] Morath SU, Pratt PD, Silvers CS, Center TD (2006) Herbivory by *Boreioglycaspis melaleucae* (Hemiptera: Psyllidae) accelerates foliar senescence and abscission in the invasive tree *Melaleuca quinquenervia* . Environmental Entomology 35: 1372–1378.

[6] Balentine KM, Pratt PD, Dray FA, Rayamajhi MB, Center TD (2009) Geographic distribution and regional impacts of *Oxyops vitiosa* (Coleoptera: Curculionidae) and *Boreioglycaspis melaleucae* (Hemiptera: Psyllidae), biological control agents of the invasive tree *Melaleuca quinquenervia* . Environmental Entomology 38: 1145–1154. 19689893 10.1603/022.038.0422

[7] Pratt PD, Morath SU, Silvers CS, Center TD (2006) Herbivory by *Boreioglycaspis melaleucae* (Hemiptera: Psyllidae) accelerates foliar senescence and abscission in the invasive tree *Melaleuca quinquenervia* . Environmental Entomology 35: 1372–1378.

[8] Franks SJ, Kral AM, Pratt PD (2006) Herbivory by introduced insects reduces growth and survival of *Melaleuca quinquenervia* seedlings. Environmental Entomology 35: 366–372.

[9] Center TD, Pratt PD, Tipping PW, Rayamajhi MB, Van TK, et al (2007) Initial impacts and field validation of host range for *Boreioglycaspis melaleucae* Moore (Hemiptera: Psyllidae), a biological control agent of the invasive tree *Melaleuca quinquenervia* (Cav.) Blake (Myrtales: Myrtaceae: Leptospermoideae). Environmental Entomology 36: 569–576. 17540066 10.1603/0046-225x(2007)36[569:iiafvo]2.0.co;2

[10] Hodkinson I (2009) Life cycle variation and adaptation in jumping plant lice (Insecta: Hemiptera: Psylloidea): a global synthesis. Journal of Natural History 43: 65–179.

[11] Retnakaran A, Ennis T, Jobin L, Granett J (1979) Scanning electron-microscopic study of wax distribution on the balsam woolly aphid, *Adelges piceae* (Homoptera, Adelgidae). Canadian Entomologist 111: 67–72.

[12] Pope RD (1983) Some aphid waxes, their form and function (Homoptera: Aphididae). Journal of Natural History 17: 489–506.

[13] Kumar V, Tewari SK, Datta RK (1997) Dermal pores and wax secretion in mealybug *Maconellicoccus hirsutus* (Hemiptera, Pseudococcidae), a pest of mulberry. Italian Journal of Zoology 64: 307–311.

[14] Smith RG (1999) Wax glands, wax production and the functional significance of wax use in three aphid species (Homoptera: Aphididae). Journal of Natural History 33: 513–530.

[15] Lucchi A, Mazzoni E (2004) Wax production in adults of planthoppers (Homoptera: Fulgoroidea) with particular reference to *Metcalfa pruinosa* (Flatidae). Annals of the Entomological Society of America 97: 1294–1298.

[16] Ammar E-D, Alessandro R, Hall DG (2013) Ultrastructural and chemical studies on waxy secretions and wax-producing structures on the integument of the woolly oak aphid *Stegophylla brevirostris* Quednau (Hemiptera: Aphididae). Journal of Microscopy & Ultrastructure 1:43–50.

[17] Ammar E-D, Alessandro R, Shatters RG, Hall DG (2013) Behavioral, ultrastructural and chemical studies on the honeydew and waxy secretions by nymphs and adults of the Asian citrus psyllid *Diaphorina citri* (Hemiptera: Psyllidae). PLoS ONE 8(6): e64938 doi: 10.1371/journal.pone.0064938 23762268 10.1371/journal.pone.0064938 PMC3677883

[18] Hall DG, Ammar E-D, Richardson ML, Halbert SE (2013) Asian citrus psyllid, *Diaphorina citri* (Hemiptera: Psyllidae), vector of citrus huanglongbing disease. Entomologia Experimentalis et Applicata 146: 207–223.

[19] Catling HD (1970) Distribution of psyllid vectors of citrus greening disease, with notes on biology and bionomics of *Diaphorina-citri* . FAO Plant Protection Bulletin 18: 8–15.

[20] Husain M, Nath D (1927) The citrus psylla (*Diaphorina citri*, Kuw.) [Psyllidae: Homoptera]. Memoirs of the Department of Agriculture in India, Entomological Series 10: 1–27.

[21] Brittain WH (1923) The morphology and synonymy of *Psyllia mali* Schmidberger. Proceedings of Acadian Entomological Society 8: 23–42.

[22] Waku Y (1978) Fine-Structure and Metamorphosis of wax gland cells in a psyllid insect, *Anomoneura mor*i Schwartz (Homoptera). Journal of Morphology 158: 243–273. 10.1002/jmor.1051580302 30227697

[23] Foldi I, Pearce MJ (1985) Fine-structure of wax glands, wax morphology and function in the female scale insect, *Pulvinaria regalis* Canard (Hemiptera, Coccidae). International Journal of Insect Morphology & Embryology 14: 259–271.

[24] Nelson DR, Walker GP, Buckner JS, Fatland CL (1997) Composition of the wax particles and surface wax of adult whiteflies: *Aleuroplatus coronata*, *Aleurothrixus floccosus*, *Aleurotithius timberlakei*, *Dialeurodes citri*, *Dialeurodes citrifolii*, and *Parabemisia myricae* . Comparative Biochemistry and Physiology B-Biochemistry & Molecular Biology 117: 241–251.

[25] Foldi I (1991) The wax glands in scale insects—Comparative ultrastructure, secretion, function and evolution (Homoptera, Coccoidea). Annales De La Societe Entomologique De France 27: 163–188.

[26] Douglas AE (2006) Phloem-sap feeding by animals: problems and solutions. Journal of Experimental Botany 57: 747–754. 16449374 10.1093/jxb/erj067

[27] Gullan PJ, Kosztarab M (1997) Adaptations in scale insects. Annual Review of Entomology 42: 23–50. 15012306 10.1146/annurev.ento.42.1.23

[28] Alene DC, Djieto-Lordon C, Burckhardt D. (2011). Unusual behaviour—unusual morphology: mutualistic relationships between ants (Hymenoptera: Formicidae) and *Diaphorina enderleini* (Hemiptera: Psylloidea), associated with *Vernonia amygdalina* (Asteraceae). African Invertebrates 52: 353–361.

[29] Nelson DR, Freeman TP, Buckner JS (2000) Waxes and lipids associated with the external waxy structures of nymphs and pupae of the giant whitefly, *Aleurodicus dugesii* . Comparative Biochemistry and Physiology B-Biochemistry & Molecular Biology 125: 265–278. 10.1016/s0305-0491(99)00177-7 10817914

